# Testicular torsion and climate changes in macroregions of São Paulo, Brazil

**DOI:** 10.31744/einstein_journal/2021AO5472

**Published:** 2021-03-05

**Authors:** João Roberto Paladino, Fernando Korkes, Sidney Glina

**Affiliations:** 1 Faculdade de Medicina do ABC Santo AndréSP Brazil Faculdade de Medicina do ABC, Santo André, SP, Brazil.

**Keywords:** Spermatic cord torsion, Scrotum, Climate change, Brazil

## Abstract

**Objective:**

To analyze the association between climate changes in the macroregions in the state of São Paulo and testicular torsion treated cases.

**Methods:**

The cases were selected in the Brazilian Public Health Data System Database from January 2008 to November 2016. All surgical procedure records were identified by the Hospital Admission Authorization document. Two codes were selected to process the search: testicular torsion (surgical cure code) and acute scrotum (exploratory scrototomy code). The macroregions were grouped in five areas linked to climate characteristics by International Köppen Climate Classification.

**Results:**

A total of 2,351 cases of testicular torsion were registered in the period. For the areas B, C and E (testicular torsion n=2,130) there were statistical differences found in association of testicular torsion cases and decreased temperature (p=0.019, p=0.001 and p=0.006, respectively), however, in analyses for the areas A and D statistical differences were not observed (p=0.066 and p=0.494).

**Conclusion:**

Decrease in temperature was associated with testicular torsion in three macroregions of São Paulo. The findings support the theory of cold weather like a trigger in occurrence of testicular torsion in a tropical climate region.

## INTRODUCTION

Testicular torsion is a urological emergency caused by the torsion of the spermatic cord, cutting off the testicle’ blood supply (ischemia).^(^^1^^)^ This is a rather rare event, but it has to be immediately recognized and surgically treated.^(^^2^^)^ A surgical delay can lead to the loss of the organ.^(^^3^^)^

The general clinical feature comprises of acute pain followed by testicular edema and redness in the scrotal region. Other associated symptoms may be present, like abdominal pain, nausea, and vomiting.^(^^2^^)^ It is believed that after 4 to 6 hours from the onset of the symptoms, irreversible pathophysiological mechanisms are triggered; therefore, the timing for intervention is considered to be the most critical factor in emergency care.^(^^1^^)^

Although investigative and treatment guidelines are well established, the etiology of this condition has not been elucidated.^(^^1^^)^ Some risk clinical factors have positively been described in retrospective studies, such as short-lasting pain, high testicular position, scrotal skin changes and testicular hypermobility due to abnormalities in testicular fixation (“bell clapper deformity”).^(^^3^^)^ Some other studies have revealed significantly related events, like the rapid increase in testicular volume in cases of malignancy,^(^^3^^,^^4^^)^ local traumas,^(^^5^^)^ or the performance of certain physical activities – cycling in particular –, during which the organ can easily rotate around its axis due to the movements of the legs. The cremasteric reflex is hyperactivated by the physical strain or the cold airflow.^(^^6^^)^

Differences of local temperature, including seasonal climatic variations, have been associated with a higher incidence of testicular torsion in studies worldwide.^(^^7^^,^^8^^)^ The mechanism of testicular torsion in testis with abnormal fixation of the testicle to the tunica vaginalis can be trigger by thermal differences, especially in decreased temperatures, resulting in an asymmetric contraction of muscle fibers, which complies with the hypothesis of hyperactive cremasteric effect as a facilitator for torsion.^(^^1^^,^^9^^,^^10^^)^ Positive associations, with a significant number of testicular torsion cases related to low temperatures^(^^7^^,^^11^^,^^12^^)^ as well as the lack of association^(^^3^^,^^13^^)^ have been described in the literature.

A previous study of Korkes et al., identified 21,289 countrywide hospital admissions for the treatment of testicular torsion between the years 1992 and 2010.^(^^14^^)^ Interestingly, the highest number of cases was reported in the coldest months (p=0.002). Statistically significant differences could be observed in the Southeastern and Southern regions of the country (p<0.001).^(^^14^^)^ Another statewide Brazilian study on the association of testicular torsion with cold temperature has investigated immediate and delayed effects of atmospheric temperature in the incidence of testicular torsion.^(^^15^^)^ Therefore, the relevance of this finding justified the performance of the current study, aiming at a more thorough investigation in the macroregions of the state of São Paulo.

## OBJECTIVE

To analyze the association between climate changes in the macroregions in the state of São Paulo and testicular torsion treated cases.

## METHODS

The main data source was the *Departamento de Informática do Sistema Único de Saúde* (DATASUS), available in electronic format,^(^^16^^)^ from 2008 to 2016. It supplied all the surgical procedure records through the Hospital Admission Authorizations, which included the surgical procedure codes for testicular torsion (surgical cure: TUSS code 31203108) and acute scrotum (exploratory scrototomy: TUSS code 31203035) registered within the state of São Paulo, Brazil.

Cases of extravaginal testicular torsion, namely occurrences of intrauterine torsion that result in a missing testicle at birth, were excluded. Therefore, testicular torsion mentioned in the current study refers to intravaginal torsion cases only.

Access to monthly testicular torsion occurrences for the selected period was obtained from DATASUS; however, the exact day of each event was not available for consultation. The number of testicular torsion cases was then tabulated according to the monthly occurrences between January 2008 and November 2016, which were later used for the analysis with local temperature data.

To calculate the rates of testicular torsion cases, the mean male population who made use of Basic Health Units of *Sistema Único de Saúde* (SUS) during the studied period was taken into account according to the data supplied by the Brazilian Institute of Geography and Statistics (IBGE - *Instituto Brasileiro de Geografia e Estatística*) The mean of annual total population divulged by IBGE between the years 2008 and 2016 (38,899,506 inhabitants) was considered for calculation.^(^^17^^)^ To determine the male population, the female/male ratio of 1.111 was used (mean of the state of São Paulo, according to IBGE). Male SUS users were calculated taking into consideration that 59% of this population did not have a supplementary healthcare plan over the study period.^(^^18^^)^

**Figure 1:**
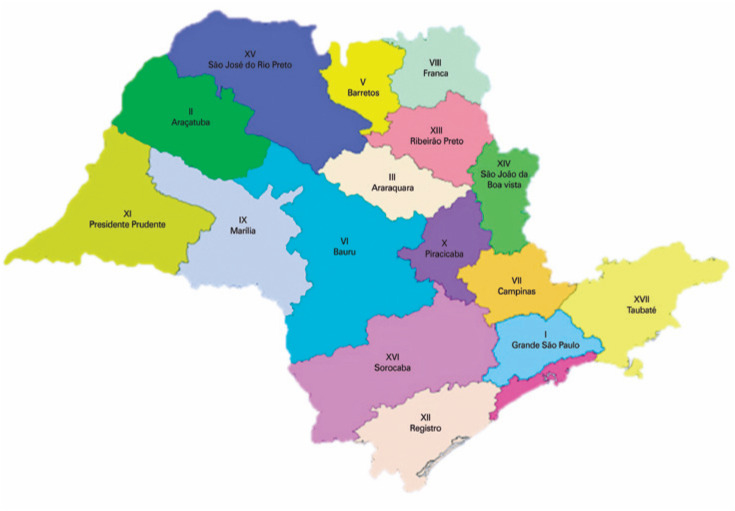
Division of São Paulo Health Departments

Source: São Paulo. Governo do Estado. Secretaria de Estado da Saúde. Regionais de Saúde. São Paulo: Secretaria de Estado da Saúde; 2012 [citado 2021 Jan 29]. Disponível em: http://www.saude.sp.gov.br/ses/institucional/departamentos-regionais-de-saude/regionais-de-saude

DATASUS supplies the data about sites of occurrence according to the administrative division of the Health State Secretariat of São Paulo in Regional Health Departments (DRS), complying with decree #51,433 published in the Official Gazette of the State of São Paulo dated from December 28, 2006. Through this decree, the state was divided into 17 DRS, which are responsible not only for the coordination of the activities of the Health State Secretariat in a regional scope but also for the promotion of intersectional articulation with municipalities and civil society organizations.^(^^19^^)^ The numbers of testicular torsion cases were tabulated according to the DRS within the studied period. Data on the local population for each DRS throughout the years were tabulated according to IBGE records and later used for the calculation of density of testicular torsion cases per region in the state. Figure 1 shows the state division into DRS.

Data on the temperature in each location were obtained from the Agrometeorology Information Center (CIIAGRO - *Centro Integrado de Informações Agrometeorológicas*)^(^^20^^)^ in São Paulo. An index city was selected for each main climate type in the state, respecting the boundaries of each DRS, not only to ensure that the sample of temperature data would be representative and comprehensive in regard to the major climate types, but also to establish the correlation between the available data from places with testicular torsion occurrences. The choice of an index city depended on the existence of a meteorological station that generated the data records, a fact that limited the selection of cities. Many times there was only one choice in the area limited by one main climate type; however, whenever there were two or more cities with a meteorological station, the city with the biggest population, based on the data supplied by the IBGE, was chosen.^(^^17^^)^

**Figure 2:**
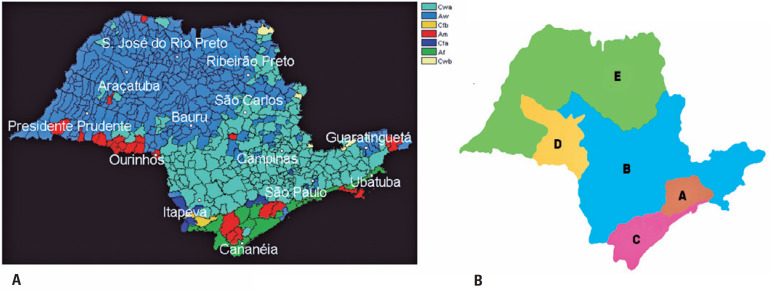
Representation of the State of São Paulo. (A) The Köppen climate classification in the state of São Paulo. (B) The division of five major areas based on the Köppen classification and the local climate correlation among the Regional Health Departments in the state of São Paulo

Source: adapted from São Paulo. Governo do Estado. Secretaria de Estado da Saúde. Regionais de Saúde. São Paulo: Secretaria de Estado da Saúde; 2012 [citado 2021 Jan 29]. Disponível em: http://www.saude.sp.gov.br/ses/institucional/departamentos-regionais-de-saude/regionais-de-saude

According to the Köppen climate classification,^(^^21^^)^ based on the rainfall and temperature monthly data, São Paulo encompasses seven distinct climate types, and most of them correspond to a wet climate. The Cwa climate is the predominant type in the biggest area, which includes all the central area of the state. It consists of an altitude tropical climate, with rainfall in the summer. In some mountain areas, where summer is mild, the climate is classified as Cwb. In the Northwest of the state, where the hottest temperatures are found, the climate is classified as Aw, *i.e*., a rainy tropical climate with dry winters. In some isolated spots, the climate type is Am, characterized by a rainy tropical climate with dry winters. In the South, there are tropical climate zones, with hot summers and no dry seasons in winter. In these zones, the Cfa type is characterized by mesothermal climate. The Serra da Mantiqueira and Serra do Mar are the highest areas, with mild summers and rainy days throughout the year. The climate type is Cbf, characterized by milder summers. The coastal strip is classified as Af, an area of a rainy tropical climate, with no dry season. Figure 2A shows the Köppen climate classification in the state of São Paulo.

Finally, five major regions of the study were established based on the Köppen classification and the local climate correlation among the DRS. For each region, an index city was chosen as shown below:

– Area A: DRS I (Great São Paulo); index city: Guarulhos; climate type: Cwa.
– Area B: DRS VI, VII, X, XIV and XVII (Bauru, Campinas, Piracicaba, São João da Boa Vista, Sorocaba and Taubaté, respectively); index city: Campinas; climate type: Cwa.
– Area C: DRS IV and XII (Baixada Santista and Registro, respectively); index city: Santos; climate type: Af.
– Area D: DRS IX (Marília); index city: Marília; climate type: Am.
– Area E: DRS II, III, V, VIII, XI, XIII and XV (Araçatuba, Araraquara, Barretos, Franca, Presidente Prudente, Ribeirão Preto and São José do Rio Preto, respectively); index city: Ribeirão Preto; climate type: Aw.

Cwa climate was divided into two great population areas, A and B, which were represented by the index city Guarulhos and Campinas, respectively, due to the demographic importance of São Paulo's metropolitan region.

Given the low population representatively, Cfa, Cfb, and Cwb climates were grouped within the predominant climate of the DRS.

Figure 2B shows the representation of the major areas described.

According to the data supplied by CIIAGRO, the daily temperatures of the index city were tabulated over the study period. The mean temperature for each month throughout the years was calculated by using the program Excel for Windows.

For the final statistical analysis, the mean temperature of the months between the years 2008 and 2016 was divided into the categories hot and cold months about the seasons of the year. Hot months refer to January, February, March, October, November, and December, corresponding to summer and spring, whereas cold months were those from April to September, corresponding to autumn and winter in the southern hemisphere.

Given the fact the current study used the data compiled from DATASUS, an Ethics Committee register was not required according to the 466/12 resolution from the National Health Council.

### Data analysis method

Temperature data normality was assessed by the Shapiro-Wilk test, and due to normal distribution adherence (p>0.05), parametric tests were applied. To analyze the association between temperature and surgery rates for the treatment of testicular torsion in each macroregions, the *t*-Student test was employed. The significance level was set at 5%. For data analysis, the Stata 11.0 (StataCorp, L.C.) was used.

## RESULTS

Between the months of January 2008 and November 2016, a total of 2,351 cases of testicular torsion in the state of São Paulo were registered on the DATASUS system. The number of testicular torsion cases per 100,000 men who used SUS services was 21,61. Table 1 displays the data collected for analysis according to the division of areas in the state, relating the number of testicular torsion events in the studied population to temperature variations registered in the hot and cold months over the study period.

**Table 1:** Descriptive data of São Paulo state according to the areas of study between the periods of 2008 and 2016

Areas of study	Index city	Köeppen classification	Mean temperature (°C)		Number of testicular torsion cases
			Hot months	Cold months	
A	Guarulhos	Cwa	23.6	19.4	145
B	Campinas	Cwa	24.2	20.2	1.093
C	Santos	Af	25.1	21.3	191
D	Marília	Am	24.6	21.1	76
E	Ribeirão Preto	Aw	24.7	21.2	846

As to the analysis of temperature variations, in all studied areas, there was a statistical difference between the hot and cold months (p<0.05) as seen in table 2. However, regarding the analysis of temperature variations between the hot and cold months and testicular torsion occurrences in the respective areas, no statistical difference could be observed in areas A and D over the study period (p=0.066 and p=0.494 respectively). A classification as hot or cold months are linked to season in the southern hemisphere (Spring: September, October and November; Summer: December, January and February; Autumn: March, April and May; Winter: June, July and August).

**Table 2:** Grouping of cold and hot months per region associated with the number of testicular torsion cases within the studied period and the number of testicular torsion events/100,000 male basic health units users

Areas of study	Months	Temperature (°C)	Number	Torsions/100,000 men
A	Hot	23.6	59	8.59
	Cold	19.4	86	12.52
	p value	0.001	0.066	
B	Hot	24.2	474	9.39
	Cold	20.2	619	12.26
	p value	0.001	0.019	
C	Hot	25.1	71	8.53
	Cold	21.3	120	14.42
	p value	0.002	0.001	
D	Hot	24.6	35	8.11
	Cold	21.1	41	9.50
	p value	0.001	0.494	
E	Hot	24.7	364	7.21
	Cold	21.2	482	9.55
	p value	0.001	0.006	

**Figure 3:**
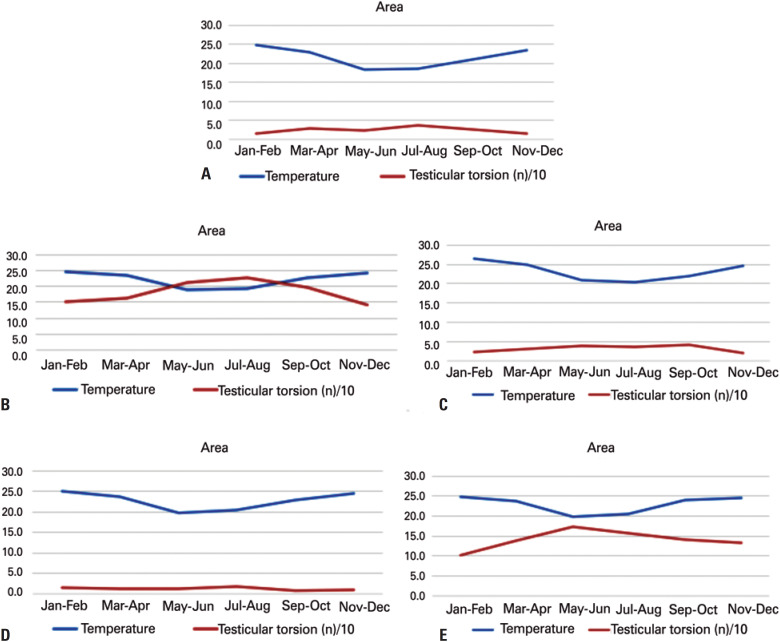
Register of mean temperatures (°C) according to the months of the year and the cumulative records of cases (x10^−1^) per month that occurred in the regions between 2008 and 2016. (A) Temperature and testicular torsion cases in area A; (B) Temperature and testicular torsion cases in area B; (C) Temperature and testicular torsion cases in area C; (D) Temperature and testicular torsion cases in area D; E) Temperature and testicular torsion cases in area E

Figure 3 shows the mean temperature registered according to the months of the year and the cumulative records of those cases that occurred in the respective areas over the study period.

## DISCUSSION

Acute testicular pain (acute scrotum) is as a medical urgency. Delayed diagnosis and an inadequate therapy may not only lead to the loss of a testicle but also result in legal implications for the urologist,^(^^22^^)^ Gaither et al.,^(^^23^^)^ in a work that studied appeal cases in court decisions on medical malpractice in the United States.

The current study has come to interesting conclusions about the theme. After analyzing 2,351 cases in São Paulo registered on DATASUS between 2008 and 2016, it could be observed that there is a correlation between testicular torsion and temperature.

Environmental temperature has been associated with variations in the incidence of testicular torsion. Cremaster muscle spasm in response to the cold weather or pain has been implied in the beginning and maintenance of testicular torsion.^(^^24^^)^ Although anatomical abnormalities are quite common in patients with testicular torsion, such as testicles with a greater horizontal axis and spermatic cord with a long intrascrotal portion, only a small rate of patients with anatomical abnormalities have testicular torsion. The cold weather, as well as the resultant cremasteric hyperactivity, can be predisposing factors for torsion in individuals with unfavorable anatomy.^(^^25^^)^

This study corroborates the findings in the literature, as shown in studies by Shukla et al.,^(^^10^^)^ and Mabogunje^(^^26^^)^ who reported an increase in testicular torsion incidence during the cold months in the United Kingdom and Nigeria respectively.

The strong points of this study are the size of the sample (2,351 cases), the long period of analysis (9 years approximately) and the relevance of the national data, which were previously published by the same group of authors.^(^^14^^)^

According to the analyzed data, despite the fact areas A and B have the same type of climate, the absolute and relative numbers of testicular torsion cases were different between each other. This can be explained by the differences in the demographic profile between the areas, such as greater population aging and a higher rate of supplementary healthcare plans in area A when compared with area B. Moreover, the improvement in healthcare conditions in area A allows for better identification of differential diagnosis, thus preventing scrotal exploration surgery due to diagnostic doubt and the over-registration of cases that do not confirm the diagnosis of testicular torsion when surgically treated.

The limitations the current study had to overcome were as follows: DATASUS database does not provide the correlation with clinical outcomes whenever patients have a differential diagnosis in the intraoperative setting, and such cases remain registered as testicular torsion events in the Hospital Admission Authorization (AIH – *Autorização de Internação Hospitalar*). The data obtained from DATASUS do not enable correlation with patients’ age group. It was not possible to access the official data from regional bases that include private healthcare services. It was not possible to associate the mean temperature of the day with event occurrences since data are registered as events/month on DATASUS.

Relevant confounding factors can also be mentioned, such as the occurrence of the following seasonal events: polar air masses that influence the temperatures in the state of São Paulo; dry air masses and high-pressure zones that result in the rising of temperature, thus affecting testicular torsion registration. However, it is important to point out that the use of monthly mean temperatures over the study period minimized the effects of such phenomena on the analyzed data. A prospective cohort study would be the ideal design for the analysis of the association between temperature and testicular torsion incidence, but the low frequency of event occurrences would make the study difficult to be carried out.

Public health policies should be developed to instruct the general population from regions with higher incidence rates to search for immediate medical help as soon as the first symptoms appear, especially in the colder months.

## CONCLUSION

A decrease in temperature was associated with testicular torsion in three macroregions of São Paulo State. The findings support the theory of cold weather like a trigger in the occurrence of testicular torsion in a tropical climate region. Strategies to prevent these events can be based on these findings.
