# Supplementary material for: Testicular torsion and climate changes in macroregions of São Paulo, Brazil
Source: Einstein (Sao Paulo). 2021 Mar 5;19:eAO5472. doi: 10.31744/einstein_journal/2021AO5472 (PMC7935086; doi:10.31744/einstein_journal/2021AO5472)
Supplement: Translation

# Translation: Torção testicular e diferenças climáticas nas macrorregiões do estado de São Paulo, Brasil

**Authors:** João Roberto Paladino, Fernando Korkes, Sidney Glina

**Affiliations:** 1 Santo AndréSP Brasil Faculdade de Medicina do ABC, Santo André, SP, Brasil.

**Keywords:** Torção do cordão espermático, Escroto, Mudança climática, Brasil

## Abstract

**Objetivo:**

Analisar a associação entre mudanças climáticas nas macrorregiões do estado de São Paulo e os casos tratados com torção testicular registrados.

**Métodos:**

Os casos foram selecionados no Banco de Dados do Sistema de Dados de Saúde Pública do Brasil de janeiro de 2008 a novembro de 2016. Todos os registros de procedimentos cirúrgicos foram identificados pelo documento de Autorização de Internação Hospitalar. Dois códigos foram selecionados para processar a busca: torção testicular (código de cura cirúrgica) e escroto agudo (código de escrototomia exploratória). As macrorregiões foram agrupadas em cinco áreas ligadas às características climáticas pela Classificação Internacional de Clima Köppen.

**Resultados:**

Foram registrados 2.351 casos de torção testicular no período. Para as áreas B, C e E (torção testicular n=2.130), foram encontradas diferenças estatísticas na associação dos casos de torção testicular e diminuição da temperatura (p=0,019, p=0,001 e p=0,006, respectivamente), mas nas análises das áreas A e D não foram observadas diferenças estatísticas (p=0,066 e p=0,494).

**Conclusão:**

A diminuição da temperatura foi associada à torção testicular em três macrorregiões de São Paulo. Os resultados apoiam a teoria do clima frio como um gatilho para ocorrência de torção testicular em uma região de clima tropical.

## INTRODUÇÃO

Torção testicular é uma emergência urológica causada pela torção do cordão espermático, cortando o suprimento sanguíneo do testículo (isquemia).^(^^1^^)^ Trata-se de um evento raro, mas que requer reconhecimento imediato e tratamento cirúrgico.^(^^2^^)^ A demora cirúrgica pode levar à perda do órgão.^(^^3^^)^

As características clínicas gerais consistem em dor aguda seguida de edema testicular e vermelhidão na região escrotal. Outros sintomas associados podem ser apresentados, como dor abdominal, náusea e vômito.^(^^2^^)^ Acredita-se que, após 4 a 6 horas do início dos sintomas, mecanismos patofisiológicos irreversíveis são desencadeados; assim, o tempo para intervenção é considerado o fator mais crítico no cuidado de emergência.^(^^1^^)^

Apesar das diretrizes investigativas e de tratamento estarem bem estabelecidas, a etiologia dessa condição não foi elucidada.^(^^1^^)^ Alguns fatores clínicos de risco têm sido positivamente descritos em estudos retrospectivos, como dor de duração curta, alta posição testicular, mudanças na pele escrotal e hipermobilidade testicular devido a anormalidades na fixação testicular (“badalo de sino”).^(^^3^^)^ Alguns outros estudos têm revelado eventos significativamente relacionados, como o rápido aumento do volume testicular em casos de malignidade,^(^^3^^,^^4^^)^ traumas locais^(^^5^^)^ ou no desempenho de certas atividades físicas – ciclismo em particular –, em que órgão pode ser facilmente circulado em seu eixo devido aos movimentos das pernas. O reflexo cremastérico é hiperativado por meio do esforço físico ou do ar mais frio.^(^^6^^)^

Diferenças na temperatura local, incluindo variações climáticas sazonais, têm sido associadas com alta incidência de torção testicular em estudo em diversas partes do mundo.^(^^7^^,^^8^^)^ O mecanismo de torção testicular nos testículos com fixação anormal na túnica vaginal pode ser desencadeado por diferenças termais, especialmente na redução de temperaturas, resultando em contração assimétrica das fibras musculares, que corrobora a hipótese do reflexo cremastérico hiperativo como um facilitador para a torção.^(^^1^^,^^9^^,^^10^^)^ As associações positivas, com número significante de casos de torção testicular relacionado a baixas temperaturas,^(^^7^^,^^11^^,^^12^^)^ bem como a falta de associação,^(^^3^^,^^13^^)^ têm sido descritas na literatura.

Estudo anterior de Korkes et al., identificou 21.289 admissões para tratamento de torção testicular entre os anos de 1992 e 2010.^(^^14^^)^ Curiosamente, o maior número de casos foi relatado nos meses mais frios (p=0,002). Diferenças estatisticamente significantes poderiam ser observadas nas Regiões Sul e Sudeste do país (p<0,001).^(^^14^^)^ Outro estudo em estado brasileiro conduzido sobre a associação de torção testicular com temperatura fria investigou os efeitos posteriores imediatos da temperatura atmosférica no incidente de torção testicular.^(^^15^^)^ Desse modo, a relevância desses achados justificam a realização do nosso estudo, que tem como objetivo investigar a torção testicular de forma mais ampla nas macrorregiões do estado de São Paulo.

## OBJETIVO

Analisar a associação entre mudanças climáticas nas macrorregiões do estado de São Paulo e os casos tratados com torção testicular registrados.

## MÉTODOS

A principal fonte de dados foi o Departamento de Informática do Sistema Único de Saúde (DATASUS), disponível em formato eletrônico.^(^^16^^)^ Foram consultados dados de 2008 a 2016. Esse sistema disponibilizou todos os procedimentos cirúrgicos registrados por meio da Autorização de Internação Hospitalar, que incluiu os códigos do procedimento cirúrgico para torção testicular (cura cirúrgica: código TUSS 31203108) e escroto agudo (escrotomia exploratória: código TUSS 31203035) registrados no estado de São Paulo.

Os casos de torção testicular extravaginal, nomeados “ocorrência de torção intrauterina”, que resulta na ausência de testículo ao nascer, foram excluídos. Assim, a torção testicular mencionada no presente estudo refere-se somente aos casos de torção intravaginal.

O acesso mensal às ocorrências de torção testicular para o período selecionado foi obtido do DATASUS, porém, o dia exato de cada evento não estava disponível para consulta. O número de casos de torção testicular foi, então, tabulado de acordo com as ocorrências mensais entre janeiro de 2008 e novembro de 2016, que foram mais tarde utilizadas para análise com dados locais de temperatura.

Para calcular a taxa de casos de torção testicular, a média da população masculina que utilizou as Unidades Básicas de Saúde do Sistema Único de Saúde (SUS) durante o período estudado foi considerada de acordo com dados disponibilizados pelo Instituto Brasileiro de Geografia e Estatística (IBGE). A média da população anual total divulgada pelo IBGE entre os anos de 2008 e 2016 (38.899.506 habitantes) foi considerada para cálculo.^(^^17^^)^ Para determinar a população masculina, a razão homem/mulher de 1,111 foi utilizada (média do estado de São Paulo, de acordo com IBGE). Os usuários do sexo masculino foram calculados considerando que 59% da população não possuía plano de saúde suplementar ao longo do período do estudo.^(^^18^^)^

O DATASUS disponibilizava os locais de ocorrência de acordo com a divisão administrativa da Secretária da Saúde do Estado de São Paulo em Departamentos Regionais de Saúde (DRS), de acordo com decreto 51.433 publicado no Diário Oficial do Estado em 28 de dezembro 28 de 2006. Por meio desse decreto, o estado foi dividido em 17 DRS, que são responsáveis não apenas por coordenar as atividades da Secretária da Saúde no escopo regional, mas também pela promoção de uma articulação intersecional com municípios e organizações da sociedade civil.^(^^19^^)^ O número de casos de torção testicular foi tabulado de acordo com os DRS no período estudado. Os dados da população local para cada DRS ao longo dos anos foram tabulados com registros do IBGE e, mais tarde, utilizados para cálculo da densidade dos casos de torção testicular por região do estado. A figura 1 mostra a divisão do estado em DRS.

**Figura 1:** Divisão de São Paulo em Departamentos de Saúde

Fonte: São Paulo. Governo do Estado. Secretaria de Estado da Saúde. Regionais de Saúde. São Paulo: Secretaria de Estado da Saúde; 2012 [citado 2021 Jan 29]. Disponível em: http://www.saude.sp.gov.br/ses/institucional/departamentos-regionais-de-saude/regionais-de-saude

Os dados da temperatura em cada localização foram obtidos a partir do Centro Integrado de Informações Agrometeorológicas (CIIAGRO),^(^^20^^)^ em São Paulo (SP). Uma cidade índice foi selecionado para cada tipo principal de clima no estado, respeitando os limites de cada DRS, não somente para assegurar que a amostra dos dados de temperatura seria representativa e compreendida em relação aos principais tipos de clima, mas também para estabelecer correlação entre os dados disponíveis de locais com ocorrência de torção testicular. A escolha de uma cidade índice depende da existência de uma estação meteorológica que gere registros de dados, um fato que limita a seleção das cidades. Muitas vezes há apenas uma escolha na área limitada por um único tipo climático, porém, onde há duas ou mais cidades com estações meteorológicas, foi escolhida a cidade com a maior população, baseada na data disponibilizada pelo IBGE.^(^^17^^)^

De acordo com a Classificação Climática de Köppen,^(^^21^^)^ baseada na temporada de chuvas e em dados de temperatura mensal, São Paulo envolve sete tipos de clima distintos, e a maioria deles corresponde a clima úmido. O clima Cwa é o tipo predominante na maior parte das áreas, incluindo as centrais do estado. Consiste em um clima tropical de altitude, com chuvas no verão. Em algumas áreas montanhosas, onde o verão é ameno, o clima é classificado como Cwb. No noroeste do estado, onde as temperaturas são mais quentes, o clima é classificado como Aw, que é tropical chuvoso com invernos secos. Em alguns locais isolados, o tipo de clima é Am, caracterizado como tropical chuvoso com invernos secos. No Sul, há zonas de clima tropical, com verões quentes e estações secas no inverno. Nessas zonas, o tipo Cfa é classificado como clima mesotermal. A Serra da Mantiqueira e a Serra do Mar são áreas mais altas, com verões leves e dias chuvosos ao longo do ano. O tipo de clima é Cbf, caracterizado por verões amenos. A costa é classificada como Af, uma área de clima de chuvas tropicais sem estações secas. A figura 2A mostra o Classificação Climática de Köppen no estado de São Paulo.

**Figura 2:** Representação do Estado de São Paulo. (A) Classificação climática de Köppen no estado de São Paulo. (B) Divisão das cinco principais áreas baseadas na classificação climática de Köppen e correlação entre os Departamentos Regionais de Saúde no estado de São Paulo

Fonte: adaptado de São Paulo. Governo do Estado. Secretaria de Estado da Saúde. Regionais de Saúde. São Paulo: Secretaria de Estado da Saúde; 2012 [citado 2021 Jan 29]. Disponível em: http://www.saude.sp.gov.br/ses/institucional/departamentos-regionais-de-saude/regionais-de-saude

Finalmente, cinco principais regiões do estudo foram estabelecidas baseadas na Classificação Climática de Köppen e na correlação de clima local entre os DRS. Para cada região, uma cidade índice foi escolhida:

– Área A: DRS I (Grande São Paulo); cidade índice: Guarulhos; tipo de clima: Cwa.
– Área B: DRS VI, VII, X, XIV e XVII (Bauru, Campinas, Piracicaba, São João da Boa Vista, Sorocaba e Taubaté, respectivamente); cidade índice: Campinas; tipo de clima: Cwa.
– Área C: DRS IV e XII (Baixada Santista e Registro respectivamente); cidade índice: Santos; tipo de clima: Af.
– Área D: DRS IX (Marília); cidade índice: Marília; tipo de clima: Am.
– Área E: DRS II, III, V, VIII, XI, XIII e XV (Araçatuba, Araraquara, Barretos, Franca, Presidente Prudente, Ribeirão Preto e São José do Rio Preto, respectivamente); cidade índice: Ribeirão Preto; tipo de clima: Aw.

O clima Cwa foi divido em duas grandes áreas de população, A e B, representadas por cidade índice: de Guarulhos e Campinas, respectivamente, dada a sua importância demográfica na região de São Paulo.

Considerando a baixa representatividade da população, os climas Cfa, Cfb e Cwb foram agrupados em clima predominante do DRS.

A figura 2B mostra a representação das principais áreas descritas.

De acordo com dados oferecidos pelo CIIAGRO, as temperaturas diárias das cidades índices foram tabuladas ao longo do período do estudo. A temperatura média para cada mês ao longo dos anos foi calculada por meio do uso do programa Excel para Windows.

Para análise estatística final, a temperatura média dos meses entre os anos de 2008 e 2016 foi dividida em categorias de meses frios e quentes, durante as estações do ano. Os meses quentes referiram-se a janeiro, fevereiro, março, outubro, novembro, e dezembro, correspondentes ao verão e à primavera, enquanto os meses frios foram de abril a setembro, correspondentes ao outono e ao inverno no hemisfério sul.

Pelo fato de o estudo atual utilizar dados compilados do DATASUS, não foi requerido registro no Comitê de Ética, de acordo com resolução 466/12 do Conselho Nacional de Saúde.

### Método de análise de dados

A normalidade dos dados da temperatura foi avaliada por meio do teste de Shapiro-Wilk e, devido à aderência à distribuição normal (p>0,05), testes paramétricos foram aplicados. Para analisar a associação entre temperatura e taxas cirúrgicas para o tratamento da torção testicular em cada macrorregião, o teste *t* de Student foi empregado. O nível de significante foi estabelecido em 5%. Para análise de dados, foi utilizado o Stata 11.0 (StataCorp, L.C.).

## RESULTADOS

Entre os meses de janeiro 2008 e novembro 2016, 2.351 casos de torção testicular no estado de São Paulo foram registrados no sistema do DATASUS. O número de casos de torção testicular por 100 mil homens que utilizaram o serviço do SUS foi de 21.61. A tabela 1 mostra a coleta de dados para análise de acordo com a divisão de áreas no estado, relacionando o número de eventos de torção testicular no estudo populacional às variações da temperatura registradas nos meses quentes e frios ao longo do período do estudo.

**Tabela 1:** Dados descritivos do estado de São Paulo de acordo com áreas de estudo entre os períodos de 2008 e 2016

Áreas de estudo	Cidade índice	Classificação climática de Köppen	Média de temperatura (°C)		Total de casos de torção testicular
			Meses quentes	Meses frios	
A	Guarulhos	Cwa	23,6	19,4	145
B	Campinas	Cwa	24,2	20,2	1.093
C	Santos	Af	25,1	21,3	191
D	Marília	Am	24,6	21,1	76
E	Ribeirão Preto	Aw	24,7	21,2	846

Na análise das variações de temperatura, em todas as área estudadas, houve diferença estatística entre os meses quentes e frios (p<0,05), como observado na tabela 2. Porém, em relação à análise das variações de temperaturas entre os meses quentes e frios e da ocorrência de torções testiculares nas respectivas áreas, as diferenças estatísticas não podem ser observadas nas áreas A e D ao longo do período de estudo (p=0,066 e p=0,494, respectivamente). Classificação como meses quentes e frios esteve relacionada a estação no hemisfério sul (primavera: setembro, outubro e novembro; verão: dezembro, janeiro e fevereiro; outono: março, abril e maio; inverno: junho, julho e agosto).

**Tabela 2:** Agrupamento de meses frios e quentes por região associado com número de casos de torção testicular dentre o período estudado e número de eventos de torção testicular por 100 mil usuários homens das Unidades Básicas de Saúde

Areas de estudo	Meses	Temperatura (°C)	Números	Torções /100,000 homens
A	Quente	23,6	59	8,59
	Frio	19,4	86	12,52
	Valor de p	0,001	0,066	
B	Quente	24,2	474	9,39
	Frio	20,2	619	12,26
	Valor de p	0,001	0,019	
C	Quente	25,1	71	8,53
	Frio	21,3	120	14,42
	Valor de p	0,002	0,001	
D	Quente	24,6	35	8,11
	Frio	21,1	41	9,50
	Valor de p	0,001	0,494	
E	Quente	24,7	364	7,21
	Frio	21,2	482	9,55
	Valor de p	0,001	0,006	

A figura 3 mostra a média de temperatura registrada de acordo com os meses do ano e os registros cumulativos dos casos que ocorreram nas áreas respectivas, ao longo do período do estudo.

**Figura 3:** Registro de média de temperatura (°C) de acordo com meses do ano e casos de registros cumulativos (x10^−1^) por mês que ocorrem nas regiões entre 2008 e 2016. (A) Casos de temperatura e torção testicular na Área A; (B) Casos de temperatura e torção testicular na Área B; (C) Casos de temperatura e torção testicular na Área C; (D) Casos de temperatura e torção testicular na Área D; (E) Casos de temperatura e torção testicular na Área E

## DISCUSSÃO

A dor testicular aguda (escroto agudo) é uma urgência médica. A demora no diagnóstico e a terapia inadequada podem levar não somente à perda de um testículo, mas também a implicações legais para o urologista,^(^^22^^)^ como mostra Gaither et al.,^(^^23^^)^ em estudo de casos de apelação em sentenças de processos médicos nos Estados Unidos.

Este estudo obteve conclusões interessantes sobre o tema. Após analisar 2.351 casos em São Paulo registrados no DATASUS entre 2008 e 2016, foi possível observar que há correlação entre torção testicular e temperatura.

A temperatura ambiente tem sido associada com variações no incidente de torção testicular. Espasmo do músculo *cremaster* em resposta ao clima frio ou à dor implica no começo e na manutenção da torção testicular.^(^^24^^)^ Apesar das anormalidades anatômicas serem bem comuns nos pacientes com torção testícular, como testículos com maior eixo horizontal e cordão espermáticos com larga porção intraescrotal, apenas pequena parte dos pacientes com anormalidades anatômicas tem torção testicular. O clima frio, bem como a resultante da hiperatividade cremaster, pode ser fator de predisposição à torção em indivíduos com anatomia desfavorável.^(^^25^^)^

Nosso estudo corroba os achados na literatura, como o demonstrado por Shukla et al.,^(^^10^^)^ e Mabogunje,^(^^26^^)^ que relataram aumento na incidência de torção testicular durante os meses frios no Reino Unido e na Nigéria, respectivamente.

Os pontos fortes do estudo são o tamanho da amostra (2.351 casos), o longo período de análise (9 anos, aproximadamente) e a relevância dos dados nacionais, que foram previamente publicados pelo mesmo grupo de autores.^(^^14^^)^

De acordo com dados analisados, apesar de as áreas A e B terem o mesmo tipo de clima, os números absoluto e relativo dos casos de torção testicular foram diferentes entre cada um deles. Isso pode ser explicado por variações no perfil demográfico entre as áreas, como maior população, idade e alta taxa de planos de saúde suplementar na área A em comparação com a B. Além disso, a melhora nas condições de saúde na área A permite a melhor identificação do diagnóstico diferencial, prevenindo a cirurgia de exploração escrotal, devido à dúvida no diagnóstico, e o registro excessivo de casos que não confirmaram o diagnóstico de torção testicular quando tratado cirurgicamente.

As limitações que este estudo teve de superar foram as seguintes: a base do DATASUS não disponibiliza a correlação com resultados clínicos e tampouco os pacientes têm diagnóstico diferencial no ambiente intraoperatório. Tais casos permaneceram registrados como eventos de torção testicular na Autorização de Internação Hospitalar. Os dados obtidos do DATASUS não possibilitam correlação com o grupo de idade dos pacientes. Não foi possível acessar os dados oficiais das bases regionais que incluem serviços de saúde privado. Não foi possível associar a média de temperatura do dia com ocorrências de evento, já que os dados são registrados como eventos por mês no DATASUS.

Fatores de confusão podem também ser mencionados, como ocorrência dos seguintes eventos sazonais: massas de ar polar que influencia na temperatura no estado de São Paulo e massas de ar seco e zonas de alta pressão que resultam na elevação da temperatura, afetando o registro de torção testicular. Porém, é importante pontuar que o uso de média mensal de temperaturas ao longo do período do estudo minimizou os efeitos de tal fenômeno nos dados analisados. Um estudo coorte prospectivo seria um desenho ideal para análise da associação entre temperatura e incidência de torção testicular, porém a baixa frequência da ocorrência desse evento dificultaria a realização desse estudo.

As políticas públicas devem ser desenvolvidas para instruir a população geral das regiões com taxas de incidência mais altas a buscar por apoio médico imediato, assim que os primeiros sintomas aparecem, especialmente nos meses mais frios.

## CONCLUSÃO

A diminuição na temperatura foi associada com torção testicular em três macrorregiões do estado de São Paulo. Os achados deste estudo apoiam a teoria de que climas frios são um gatilho para ocorrência da torção testicular em uma região de clima tropical. Estratégias para prevenir esses eventos podem ser baseadas nesses achados.
